# Investigation on pore structure regulation of activated carbon derived from sargassum and its application in supercapacitor

**DOI:** 10.1038/s41598-022-14214-w

**Published:** 2022-06-16

**Authors:** Shijie Li, Xiaopeng Tan, Hui Li, Yan Gao, Qian Wang, Guoning Li, Min Guo

**Affiliations:** School of Thermal Engineering, Shandong Jianzhu University, Jinan, 250101 Shandong China

**Keywords:** Biomaterials, Materials for energy and catalysis, Structural materials

## Abstract

In order to realize the effective regulation of the pore structure of activated carbon and optimize its pore structure properties as electrode material, the effects of activation temperature, activation time and impregnation ratio on the specific surface area, total pore volume and average pore diameter of activated carbon prepared by sargassum are studied by orthogonal experiment. In addition, the electrochemical properties of sargassum-based activated carbon (SAC) and the relationship between the gravimetric capacitance and specific surface area of SAC are also studied. The SACs prepared under all conditions have high specific surface area (≥ 2227 m^2^ g^−1^) and developed pore structure, in which the pore diameter of micropores mainly concentrated in 0.4 ~ 0.8 nm, the pore diameter of mesopores mainly concentrated in 3 ~ 4 nm, and the number of micropores is far more than that of mesopores. In the activation process, the impregnation ratio has the greatest effect on the specific surface area of SAC, the activation temperature and impregnation ratio have significant effect on the total pore volume of SAC, and the regulation of the average pore diameter of SAC is mainly realized by adjusting the activation temperature. The SACs exhibit typical electric double layer capacitance performances on supercapacitors, delivering superior gravimetric capacitance of 237.3 F g^−1^ in 6 mol L^−1^ KOH electrolyte system at current density of 0.5 A g^−1^ and excellent cycling stability of capacitance retention of 92% after 10,000 cycles. A good linear relationship between gravimetric capacitance and specific surface area of SAC is observed.

## Introduction

The continuous increase in the consumption of traditional fossil fuels, such as coal, oil and natural gas, has led to an increasingly serious energy crisis and environmental pollution, which has intensified the world’s demand for renewable clean energy^1–3^. The utilization of renewable clean energy, such as solar energy, wind energy and ocean energy, has been developing rapidly in recent years. The disadvantages of these renewable energy sources, including intermittence and instability, limit their application to a great extent. An efficient energy storage system must be established to make full use of the electricity generated by these renewable clean energy sources^4^. As promising energy storage devices, lithium or other metal ion batteries, fuel cells and supercapacitors have attracted a lot of attention and gained remarkable research achievements^5–8^. According to the energy storage mechanism, supercapacitors are divided into pseudo capacitors and electric double layer capacitors (EDLCs)^3,9^. EDLCs are considered to be the most competitive in high power applications due to their electrostatic energy storage mechanism. They are also characterized by fast charging and discharging speed, long cycle life, light weight, wide range of service temperature and environmental friendliness^10–12^. The electrochemical performance of EDLCs is mainly determined by its electrode materials, so exploring new electrode materials and improving electrode material characteristics, including pore structure characteristics, are usually selected to greatly improve the electrochemical performance of EDLCs^13–16^.

According to the energy storage mechanism of the electric double layer, the electric capacity of the EDLC depends on the accumulated charge on the electric double layer of the polarization electrode. The storage charge of the electrode material occurs primarily at the interface between the electrode and the electrolyte. An extremely large accessible surface area of electrolyte ions should be possessed by the electrode material so that the EDLC has the ability to store more charges^17–20^. Carbon-based materials with high specific surface area, such as activated carbon, graphene, carbon nanotube and carbon aerogel, have become the main selection objects of electrode materials for EDLCs^21–23^. Among them, activated carbon has become the most widely used electrode materials for EDLCs due to its abundant raw materials, mature preparation method, low cost and non-toxicity^24,25^.

The specific surface area of activated carbon is an important factor affecting its electrochemical performance. Theoretically, the larger the specific surface area of activated carbon is, the higher the charge accumulation capacity of electrode/electrolyte interface is, and the greater the capacitance of EDLC will be^26–28^. However, only the surface of activated carbon which is adsorbed by electrolyte ions can generate electric double layer capacitance, so not all surface areas of activated carbon can generate electric double layer capacitance^26,27,29,30^. For activated carbon used in EDLCs, ion-accessible micropores (< 2 nm in size) are primarily responsible for the capacitance by providing effective adsorption surface area for electrolyte ions, mesopores (2 ~ 50 nm) and macropores (> 50 nm) are tied intimately with the high-rate capacitive performance by providing low resistance channels for the transport of electrolyte ions to the interior surface, while the ultrafine micropores with a large contribution to the specific surface area cannot accommodate the electrolyte ions, so its existence makes almost no contribution to the capacitance performance^31,32^. Activated carbon with both high specific surface area and reasonable pore diameter distribution has greater potential to exhibit the excellent electrochemical performance. Hence, activated carbon with optimized micropores for capacitance and appropriate amount of mesopores for high-rate capacitive performance are highly desired for EDLCs.

In this research, activated carbon was prepared from sargassum by KOH activation method. In order to realize the effective regulation of the pore structure of activated carbon by adjusting the experimental parameters in the activation process, the orthogonal experimental method was adopted to design the experiment. Orthogonal experimental method is a mathematical statistical method to deal with multi factor experiments. A set of standardized tables, namely orthogonal tables, are formulated to scientifically select experimental conditions and reasonably arrange experiments. The main advantage of orthogonal experimental method is that a few representative experimental schemes can be selected from many experimental schemes. Through the analysis of the results of these experimental schemes, we can not only determine the optimal scheme, but also analyze the influence of various factors on the experimental results, so as to determine the main influencing factors^33,34^.The optimal scheme of the experiment is determined by the range analysis method of orthogonal experiment^35,36^, and the analysis of variance is used to explore the significance of the influence of experimental factors on the experimental results^37^. The effects of activation temperature, activation time and impregnation ratio on the pore structure properties of activated carbon were studied by range and variance analysis of orthogonal experiment. In addition, the characterization of activated carbon material properties including surface morphology, microcrystalline structure, surface functional groups, and electrochemical properties were also carried out.

## Experimental

### Materials

Sargassum used in this experiment was collected from Rongcheng City, Shandong Province, China, its ultimate analysis and proximate analysis are shown in Table 1. The collected sargassum was washed thoroughly and dried in a blast drying oven for 48 h at a temperature of 120 °C. After sufficient drying, the sargassum was crushed and sieved with a quick grinder and a vibrating screen respectively, the products with particle diameter less than 180 μm were obtained.

**Table 1:** Ultimate and proximate analyses of the sargassum.

Ultimate analysis (wt%) (ad)					Proximate analysis (wt%) (ad)			
C	H	O	N	S	M	A	FC	V
41.41	5.42	34.98	3.39	1.67	2.4	10.73	14.93	71.94

Where M is the moisture, A is the ash, FC is the fixed carbon, V is the volatile.

### Preparation of activated carbon

The preparation of activated carbon usually requires several processes, including carbonization, low temperature pre-activation and high temperature activation. The effects of high temperature activation factors on the pore structure properties of activated carbon were studied. L_16_(4^3^) orthogonal experiment table without interaction was adopted to arrange the experiment. The factors and levels of orthogonal experiment are shown in Table 2.

**Table 2:** Factors and levels of orthogonal experiment.

Level	Factor		
	*T*_A_ A (°C)	*t*_A_ B (min)	*R*_I_ C
Level 1	700	90	3.0:1
Level 2	750	120	3.5:1
Level 3	800	150	4.0:1
Level 4	850	180	4.5:1

Where *T*_A_ is the activation temperature, *t*_A_ is the activation time, *R*_I_ is the impregnation ratio.

Sargassum was carbonized in nitrogen atmosphere. The carbonization temperature was 600 °C, the carbonization time was 120 min, the heating rate was 5 °C min^−1^, and the nitrogen flow rate was 2 L min^−1^. The carbon products obtained from carbonization were put into nickel crucible, fully mixed with saturated KOH solution in an impregnation ratio of *R*_I_ and then put into a drying oven for a certain time at 80 °C to remove moisture. Then the mixture was put into an atmosphere muff furnace for activation. In the activation process, the nitrogen flow rate was 2 L min^−1^, the pre-activation temperature was 350 °C, the pre-activation time was 30 min, the activation temperature was *T*_A_, the activation time was *t*_A_, and the heating rate was always 5 °C min^−1^. After activation, the nickel crucible was taken out and naturally cooled to ambient temperature in nitrogen atmosphere. The obtained products were washed with deionized water at 80 °C, then pickled to neutral with 0.1 mol L^−1^ HCl solution, and finally washed with deionized water at 80 °C to remove ionic impurities. The obtained carbon products were put into a drying oven and dried at 120 °C for 12 h.

### Preparation of supercapacitor

The activated carbon, conductive graphite and binder (PTFE emulsion) were mixed according to the mass ratio of 8:1:1, an appropriate amount of anhydrous ethanol was added to obtain the slurry, and the ultrasonic treatment was carried out for 30 min by ultrasonic cell breaker. The mixture was fully mixed, then the slurry was put into the air dry oven, and the excess ethanol was evaporated at 80 °C, until the solution became thicker slurry. The round nickel foam with a diameter of 1.5 cm was evenly coated with the slurry, and the mass of active material was about 5 mg. The coated nickel foam was put in the vacuum drying oven for 12 h at 120 °C to evaporate the excess anhydrous ethanol in the electrode. The dried electrodes were put into a hydraulic press with the pressed time of 1 min and pressure of 14 MPa. The electrodes and the membrane were assembled into a supercapacitor in the order of electrode, membrane and electrode, with an electrolyte of 6 mol L^−1^ KOH solution.

### Experimental techniques

Nitrogen adsorption–desorption measurements were carried out at 77 K by employing a JW-BK132F (JWGB Sci & Tech Ltd.). Based on nitrogen adsorption–desorption isotherms, the specific surface area of activated carbons was calculated by using BET method, isotherms were employed to calculate the specific surface area at a relative pressure range of 0.05–0.3. The pore diameter distribution of mesopores and micropores was determined by BJH and HK method, respectively. To investigate the electrochemical properties of activated carbons, the cyclic voltammetry (CV) and galvanostatic charge–discharge (GCD) test were measured on an electrochemical station (CHI760E, CH Instruments). Scanning electron microscope (Supra 55, Carl Zeiss AG, Germany) was adopted to observe the surface morphology of activated carbons. The surface functional groups of activated carbon were characterized by FT-IR Spectroscopy (in the range of 4500 ~ 500 cm^−1^). The microcrystalline structure of activated carbon was characterized by powder X-ray diffraction (Rigaku D/MAX-2500PC, equipped with Cu radiation, λ = 1.5406 Ǻ) with a step size of 0.05° and angle from 10 to 90°. The voltage supplied was 50 kV with a current of 150 mA.

## Results and discussion

Sixteen groups of activated carbon samples were prepared from sargassum according to the L_16_(4^3^) orthogonal experiment, and the pore structure properties of activated carbons were characterized. The results are shown in Table 3.

**Table 3:** Experiment and results of orthogonal design.

Number	*T*_A_ A (°C)	*t*_A_ B (min)	*R*_I_ C	*D*_Ave_ (nm)	*S*_BET_ (m^2^ g^−1^)	*V*_Tot_ (cm^3^ g^−1^)
1	700	90	3.0: 1	2.47	2577	1.04
2	700	120	3.5: 1	2.56	2422	1.32
3	700	150	4.0: 1	2.45	2862	1.62
4	700	180	4.5: 1	2.64	2509	1.24
5	750	90	3.5: 1	2.74	2458	1.45
6	750	120	3.0: 1	1.69	2550	1.45
7	750	150	4.5: 1	2.69	3321	2.16
8	750	180	4.0: 1	2.97	2735	2.34
9	800	90	4.0: 1	3.23	3228	2.91
10	800	120	4.5: 1	2.64	2901	1.77
11	800	150	3.0: 1	2.82	2321	1.51
12	800	180	3.5: 1	3.01	3155	2.37
13	850	90	4.5: 1	2.87	2674	2.25
14	850	120	4.0: 1	3.29	3362	3.08
15	850	150	3.5: 1	2.95	2528	2.18
16	850	180	3.0: 1	2.96	2227	1.64

Where *D*_Ave_ is the average diameter, *S*_BET_ is the specific surface area, *V*_Tot_ is the total pore volume.

The prepared activated carbons are marked as AC_1_ ~ AC_16_ according to the serial number. As can be seen from Table 3 that all SACs have high specific surface area (≥ 2227 m^2^ g^−1^) and large total pore volume (≥ 1.69 cm^3^ g^−1^), of which the maximum specific surface area is 3362 m^2^ g^−1^ and the maximum total pore volume is 3.08 cm^3^ g^−1^, indicating that SAC has great potential in many applications such as adsorption, gas storage and supercapacitors.

### Effect of experimental factors on specific surface area of SAC

#### Range analysis of specific surface area

**Figure 1:**
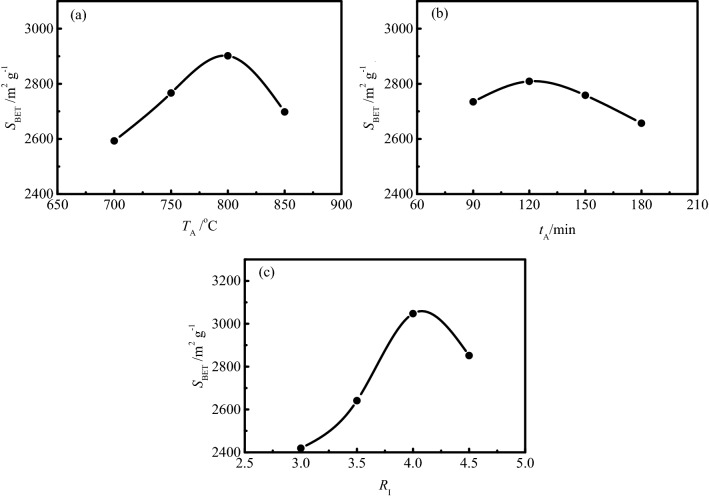
Effects of experimental factors on the specific surface area of SAC.

The range analysis of specific surface area is shown in Table 4. *K*_i_ (i = 1, 2, 3, 4) is the sum of the specific surface area value at a certain level in Table 3, *k*_*i*_ is the average value of *K*_i_, and *R* is the range. It can be seen from Table 4 that the range of specific surface area is *R*_C_ > *R*_A_ > *R*_B_, which indicates that the impregnation ratio has the greatest effect on the specific surface area of SAC, followed by activation temperature and activation time. The detailed effect of experimental factors on the specific surface area is shown in Fig. 1.

**Table 4:** The range analysis of specific surface area.

	*T*_A_ A (°C)	*t*_A_ B (min)	*R*_I_ C	*S*_BET_ (m^2^ g^-1^)
*K*_1_	10,370	10,937	9675	
*K*_2_	11,064	11,235	10,563	
*K*_3_	11,605	11,032	12,187	∑ = 43,830
*K*_4_	10,791	10,626	11,405	∑/16 = 2739
*k*_1_	2593	2734	2419	
*k*_2_	2766	2809	2641	
*k*_3_	2901	2758	3047	
*k*_4_	2698	2657	2851	
*R*	308	152	628

It can be seen from Fig. 1a that the specific surface area of SAC shows a trend of gradual increase with the increase of activation temperature until the temperature reaches 800 °C, after which the specific surface area decreases significantly with the continuous increase of temperature. With the increase of activation temperature, more energy is supplied to the activation reaction, which makes more carbon atoms at the active sites react with the activator and generate pore structure, resulting in the specific surface area of activated carbon gradually increasing. As the intermediate product of the reaction between carbonization product and activator in the activation process, metal potassium is formed and converted into potassium vapor when the activation temperature exceeds 800 °C (the boiling point of potassium is 762 °C). Potassium vapor enters the original pores and passes through the graphite microcrystalline layers, resulting in the formation of new pore structures^38^. However, the ablation of activated carbon occurs with the continuous increase of activation temperature, which leads to the collapse of pore wall and the development of larger pores. The decrease of the number of micropores and the increase of the average pore diameter resulted in the decrease of the specific surface area of activated carbon^39^.

The effect of activation time on specific surface area of SAC is shown in Fig. 1b. With the increase of activation time, more and more carbon atoms at the active sites react with KOH activator to generate pore structure, resulting in the specific surface area of activated carbon gradually increasing with the increase of activation time until the time reaches 120 min. With the continuous increase of activation time, the carbon atoms on the carbon skeleton are continuously consumed, resulting in the collapse of pore wall and the decrease of specific surface area.

It can be seen from Fig. 1c that the specific surface area of SAC increases dramatically with the increase of impregnation ratio until the impregnation ratio reaches 4.0:1, the average specific surface area of four groups of activated carbon prepared at this impregnation ratio even exceeds 3000 m^2^ g^−1^. With the increase of impregnation ratio, more and more available activators are provided to the activation reaction, and the consumption of carbon atoms at the active sites increases continuously, resulting in the increase of pore structure. However, with the continuous increase of the impregnation ratio, a trend of decrease of specific surface area appears. The carbon atoms at the active sites in the carbonized products are completely consumed with the continuous supply of activator. The excess activator reacts with the carbon atoms initially involved in the formation of pore structure, resulting in the excessive ablation of carbon materials and the increase of pore diameter, which makes a great contribution to the rapid reduction of the specific surface area of activated carbon^40^.

#### Variance analysis of specific surface area

The variance analysis of specific surface area is shown in Table 5.

**Table 5:** The variance analysis of specific surface area.

SV	*SS*	*df*	*MS*	*F*	Effect
A	200,869.25	3	66,956.4	0.5	Not significant
B	48,217.25	3	16,072.4	0.1	Not significant
C	878,090.75	3	292,696.9	2.1	Not significant
Error	833,168.50	6	138,861.4		

F_0.01_(3, 6) = 9.78; F_0.05_(3, 6) = 4.76; F_0.1_(3, 6) = 3.29.

Where SV is the source of variation, *SS* is the sum of square, *df* is the degree of freedom, *MS* is the mean square.

According to the results of variance analysis, the effects of activation temperature, activation time and impregnation ratio on the specific surface area of SAC are not significant. Especially the activation temperature and activation time, their effect on the specific surface area of SAC is negligible. Therefore, the adjustment of impregnation ratio is mainly selected to realize the regulation of specific surface area of SAC. While the activation temperature and activation time can be selected arbitrarily within the range of meeting the experimental requirements.

### Effect of experimental factors on total pore volume of SAC

#### Range analysis of total pore volume

The range analysis of total pore volume is shown in Table 6.

**Table 6:** The range analysis of total pore volume.

	*T*_A_ A (°C)	*t*_A_ B (min)	*R*_I_ C	*V*_Tot_ (cm^3^ g^−1^)
K_1_	5.22	7.64	5.64	
K_2_	7.40	7.62	7.32	
K_3_	8.55	7.47	9.95	
K_4_	9.15	7.59	7.41	∑ = 30.32
k_1_	1.30	1.91	1.41	∑/16 = 1.90
k_2_	1.85	1.90	1.83	
k_3_	2.14	1.87	2.49	
k_4_	2.29	1.90	1.85	
R	0.98	0.04	1.08	

**Figure 2:**
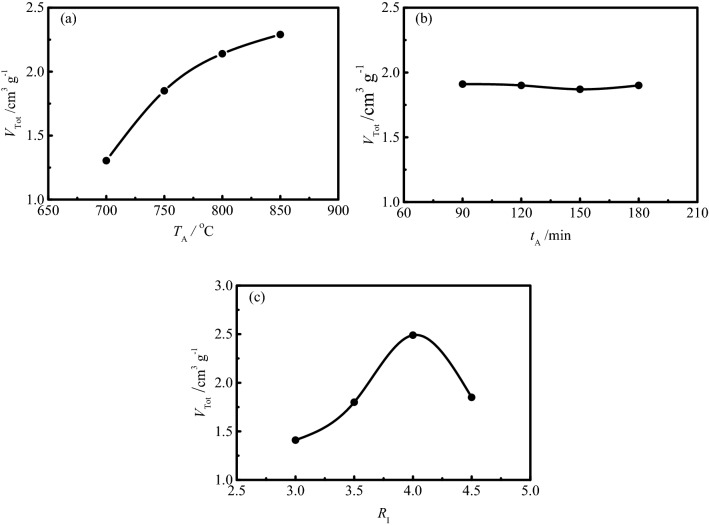
Effects of experimental factors on the total pore volume of SAC.

It can be seen from Table 6 that the range of total pore volume is *R*_C_ > *R*_A_ > *R*_B_, which indicates that the impregnation ratio has the greatest effect on the total pore volume of SAC, followed by the activation temperature, while the activation time has little effect on the total pore volume. The detailed effect of experimental factors on the total pore volume is shown in Fig. 2.

The effect of activation temperature on the total pore volume of SAC is shown in Fig. 2a. As the activation temperature increases, more energy is supplied to the activation reaction, resulting in more and more carbon atoms at the active sites being consumed to generate pore structure, eventually leading to an increase in total pore volume. In addition, the ablation of activated carbon caused by the high activation temperature leads to the collapse of the pore wall and the enlargement of the pore diameter, which also contributes to the increase of the total pore volume of activated carbon.

It can be seen from Fig. 2b that the effect of activation time on the total pore volume of SAC is negligible, indicating that the activation reaction has basically completed when the activation time reaches 90 min.

The effect of impregnation ratio on the total pore volume of SAC is shown in Fig. 2c. With the increase of impregnation ratio, the total pore volume of activated carbon increases dramatically until the impregnation ratio reaches 4.0:1, which is results from the deepening of activation degree and the development of pore structure with the increase of the impregnation ratio. However, a dramatic decrease of total pore volume is observed with the continuous increase of the impregnation ratio, which is due to the fact that very severe activation conditions mainly destroy not only the micropores but also some of the mesopores, eventually leading to the decrease of the total pore volume of activated carbon^41^.

#### Variance analysis of total pore volume

The variance analysis of total pore volume is shown in Table 7.

**Table 7:** The variance analysis of total pore volume.

SV	*SS*	*df*	*MS*	*F*	Effect
A	2.252	3	0.75	6.10	Significant
B	0.004	3	0.001	0.01	Not Significant
C	2.369	3	0.790	6.42	Significant
Error	0.739	6	0.123		

F_0.01_(3, 6) = 9.78; F_0.05_(3, 6) = 4.76; F_0.1_(3, 6) = 3.29.

According to the results of variance analysis, the effect of impregnation ratio and activation temperature on the total pore volume of SAC is significant, while the effect of activation time on the total pore volume of SAC is not significant. Therefore, the adjustment of impregnation ratio and activation temperature is mainly selected to realize the regulation of total pore volume of SAC. While the activation time can be selected arbitrarily within the range of meeting the experimental requirements.

### Effect of experimental factors on average pore diameter of SAC

#### Range analysis of average pore diameter

The range analysis of average pore diameter is shown in Table 8.

**Table 8:** The range analysis of average pore diameter.

	*T*_A_ A (°C)	*t*_A_ B (min)	*R*_I_ C	*D*_Ave_ (nm)
K_1_	10.12	11.31	9.94	
K_2_	10.09	10.18	11.26	
K_3_	11.70	10.91	11.94	
K_4_	12.07	11.58	10.84	∑ = 43.98
k_1_	2.53	2.83	2.49	∑/16 = 2.75
k_2_	2.52	2.55	2.82	
k_3_	2.93	2.73	2.99	
k_4_	3.02	2.90	2.71	
R	0.5	0.35	0.5	

**Figure 3:**
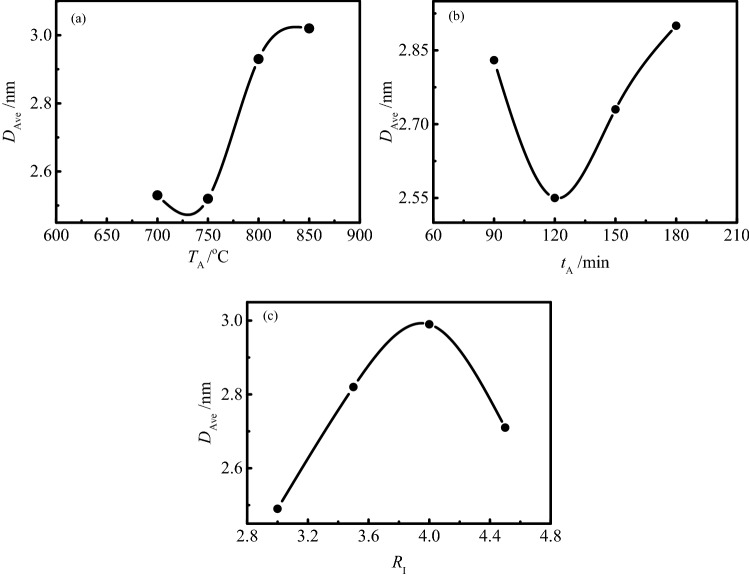
Effects of experimental factors on the average pore diameter of SAC.

It can be seen from Table 8 that the range of average pore diameter is *R*_A_ = *R*_C_ > *R*_B_, which indicates that the degree of effect of impregnation ratio and activation temperature on the average pore diameter of activated carbon is similar, and higher than that of activation time on the average pore diameter. The detailed effect of experimental factors on the average pore diameter is shown in Fig. 3.

The effect of activation temperature on the average pore diameter of SAC is shown in Fig. 3a. With the increase of activation temperature, the average pore diameter of activated carbon always presents an increasing trend. The increase in the activation temperature allows more energy to be supplied to the activation reaction, which leads to the carbon atoms at the active sites with relatively high activation energy can also get enough energy to participate in the activation reaction, so as to increase the average pore diameter of activated carbon. In addition, the pore wall collapse caused by ablation of activated carbon will also increase the average pore diameter of activated carbon.

Figure 2b shows the effect of activation time on the average pore diameter of SAC. The average pore diameter of activated carbon decreased significantly with the activation time increased from 90 to 120 min. At this stage, although the specific surface area and pore structure of activated carbon increase with the increase of activation time, the increased pore structure is mainly the ultrafine micropores with pore diameter less than 0.5 nm, which leads to the decrease of the average pore diameter of activated carbon. With the continuous increase of activation time, the increasing trend of the average pore diameter of activated carbon is observed. The continuous increase of activation time leads to the deepening of activation reaction, more carbon atoms on the pore wall participate in the activation reaction and are consumed, which leads to the further development of activated carbon pore structure and the increase of the average pore diameter.

It can be seen from Fig. 3c that the average pore diameter of activated carbon gradually increases with the increase of the impregnation ratio, except when the impregnation ratio exceeds 4.0:1, which results from the fact that the excessive impregnation ratio begins to destroy the textural development^41^.

#### Variance analysis of average pore diameter

The variance analysis of average pore diameter is shown in Table 9.

**Table 9:** The variance analysis of average pore diameter.

SV	*SS*	*df*	*MS*	*F*	Effect
A	0.809	3	0.270	3.33	Significant
B	0.278	3	0.093	1.15	Not significant
C	0.525	3	0.175	2.16	Not significant
Error	0.485	6	0.081		

F_0.01_(3, 6) = 9.78; F_0.05_(3, 6) = 4.76; F_0.1_(3, 6) = 3.29.

According to the results of variance analysis, the effect of activation temperature on the average pore diameter of SAC is significant, while the effect of activation time and impregnation ratio on the average pore diameter of SAC is not significant. Therefore, the adjustment of activation temperature is mainly selected to realize the regulation of average pore diameter of SAC. While the activation time and impregnation ratio can be selected arbitrarily within the range of meeting the experimental requirements.

### Material characterizations of SACs

#### Pore structure properties of SACs

**Figure 4:**
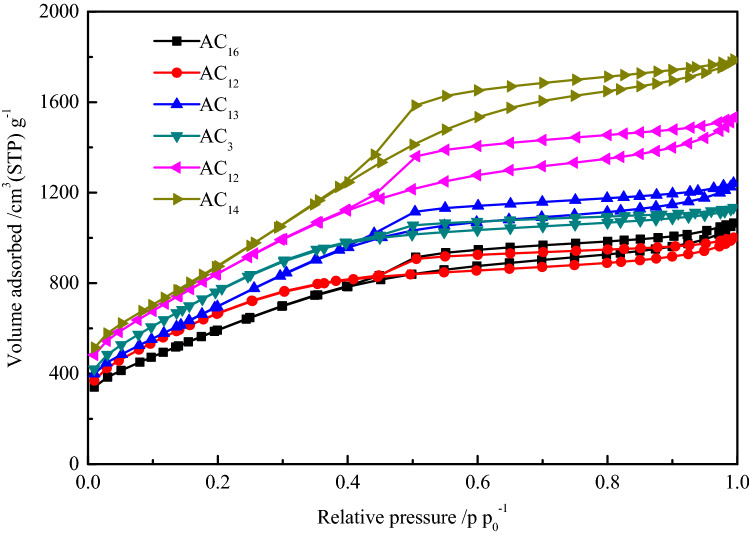
Nitrogen adsorption–desorption isotherms of SACs.

Based on the fact that all SACs have high specific surface area, reasonable pore diameter distribution can further improve the electrochemical performance of activated carbons. Nitrogen adsorption–desorption method was carried out to characterize the pore diameter distribution of activated carbon. In order to investigate the relationship between the gravimetric capacitance and specific surface area of activated carbon, SACs with specific surface areas of 2227(AC_16_), 2422(AC_2_), 2674(AC_13_), 2862(AC_3_), 3155(AC_12_) and 3362 m^2^ g^−1^(AC_14_) were selected for characterization. The nitrogen adsorption–desorption isotherms of six groups of SACs are shown in the Fig. 4.

**Figure 5:**
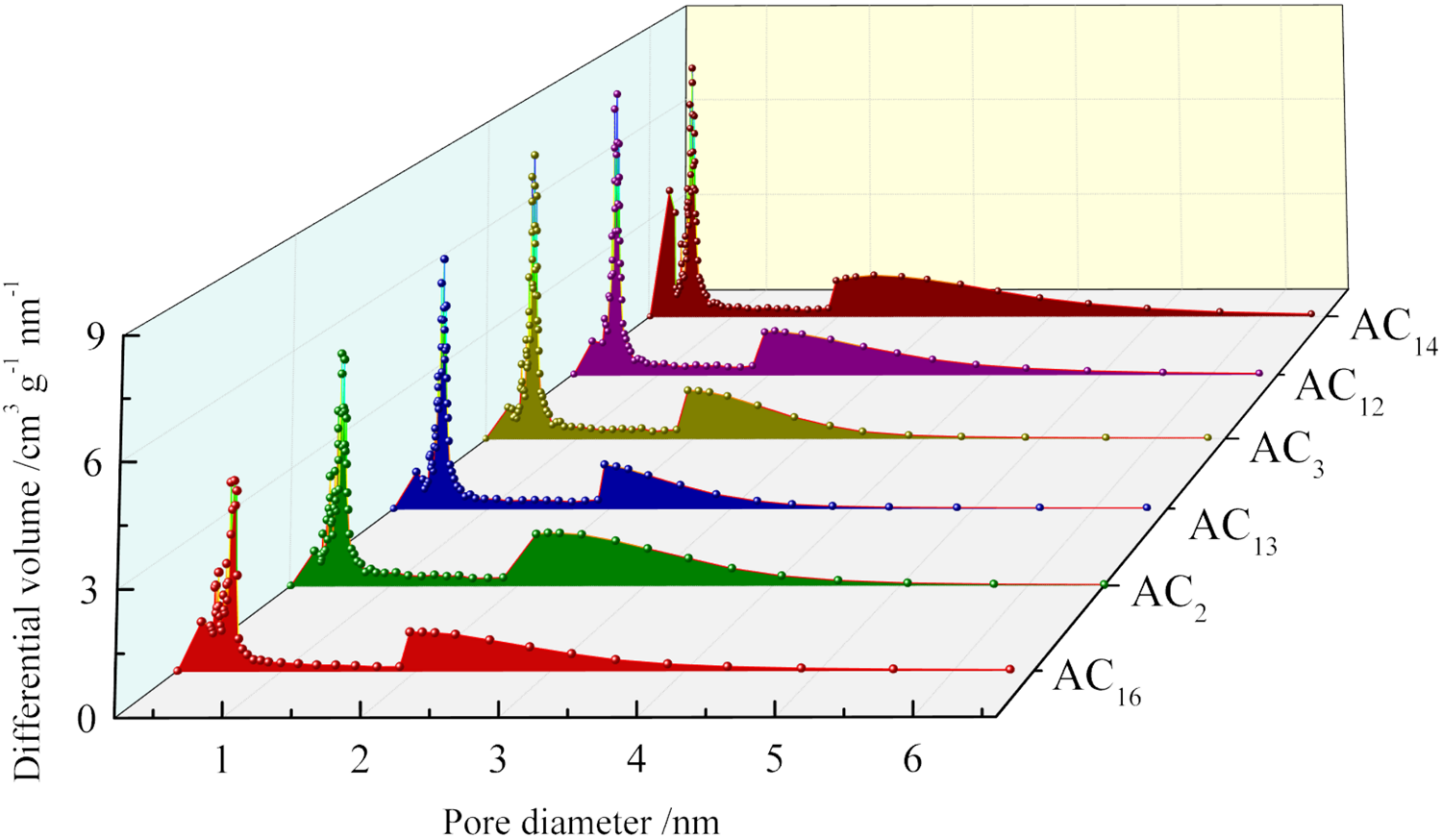
Pore diameter distribution of SACs.

Although the nitrogen adsorption capacity of the six groups of SACs is different, their adsorption–desorption isotherms show similar characteristics and all conform to type IV curve. At the low relative pressure stage, nitrogen adsorption mainly occurs in the microporous structure of activated carbon. An obvious characteristic of SAC is that the adsorption capacity of nitrogen is very large in the low relative pressure stage, which indicates the existence of a large number of microporous structures in SAC. With the increase of relative pressure, the nitrogen adsorption changes from monolayer adsorption to multilayer adsorption. In the whole process of nitrogen adsorption, the adsorption isotherm of the SACs shows a trend of continuous increase rather than the appearance of horizontal platform, indicating the existence of mesoporous structure in activated carbons. Another obvious characteristic of the adsorption–desorption isotherm of SAC is the existence of hysteresis loop. The appearance of hysteresis loop in type IV adsorption isotherm represents capillary condensation in mesopores or macropores, the hysteresis loop is closed at the relative pressure of p/p_0_ = 0.4, indicating the existence of smaller mesopores in activated carbon. The pore diameter distribution of SACs is shown in Fig. 5

.

The pore diameter distribution of all SACs is relatively concentrated, and the pore diameter is almost all distributed within 4 nm, in which the micropore diameter is mainly concentrated in 0.4 ~ 0.8 nm, the mesopore diameter is mainly concentrated in 2 ~ 4 nm, and the number of micropores is significantly higher than that of mesopores. The pore structure with a diameter of 0.5 ~ 1 nm can provide a large amount of effective internal surface area for the adsorption of electrolyte ions, while the pore structure with a diameter of 2 ~ 4 nm can provide a low resistance transport channel for ion transport into the inner surface area^42–45^. According to the nitrogen adsorption–desorption data, most of the pore diameters of SAC are in the range of 0.4 ~ 0.1 nm and 2 ~ 4 nm. Therefore, SAC has great potential to show excellent electrochemical performance when used in supercapacitors.

#### Surface morphology of SACs

**Figure 6:**
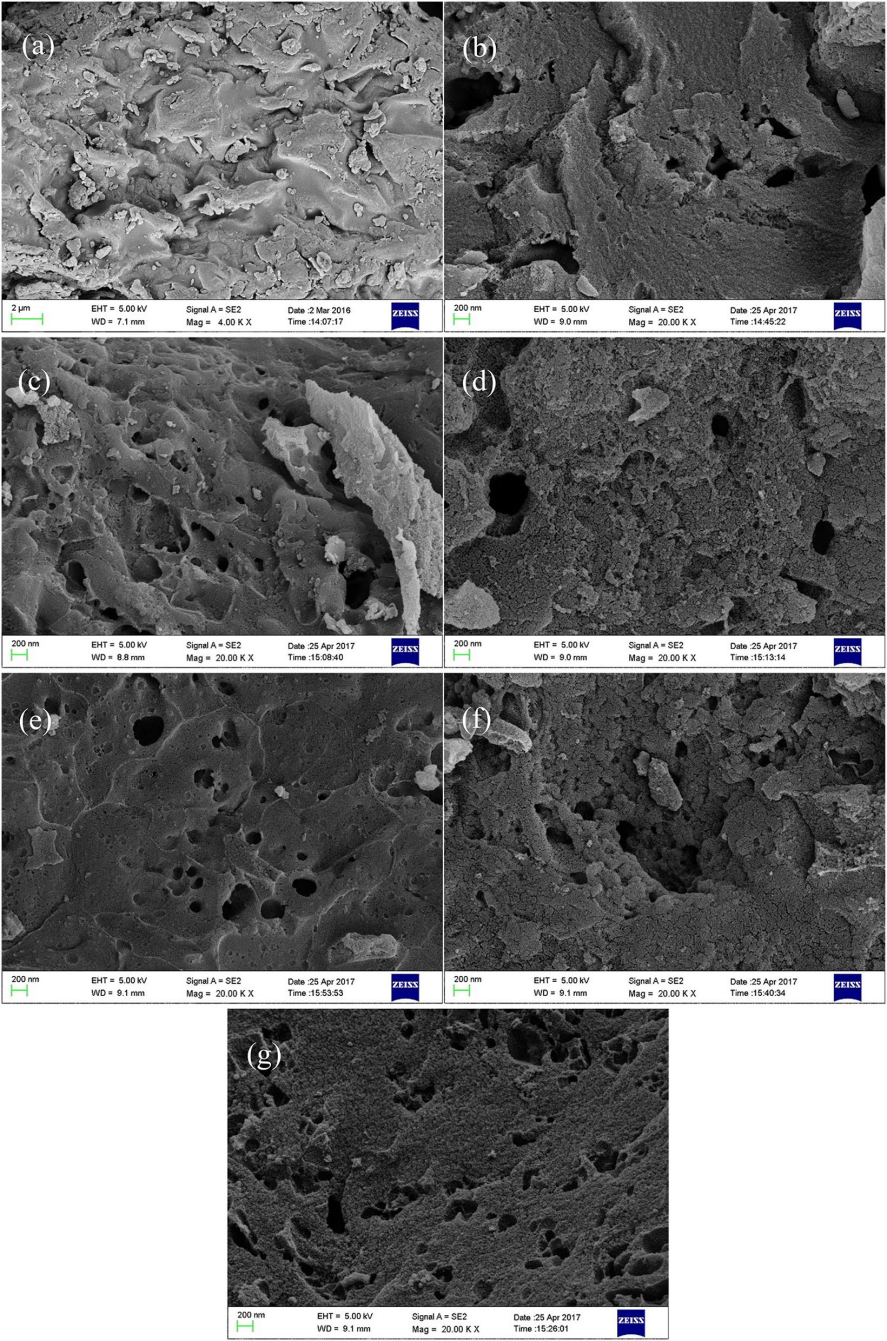
SEM of sargassum and SACs. (**a**): Sargassum; (**b**): AC_16_; (**c**): AC_2_; (**d**): AC_13_; (**e**): AC_3_; (**f**): AC_12_; (**g**): AC_14_.

The surface morphology of sargassum raw material and six groups of SACs at × 20,000 magnification is shown in Fig. 6. A significant difference between raw material and activated carbon can be observed from Fig. 6, the surface of the raw material is smooth and tidy except for some debris produced in the crushing process, and the existence of pore structure is hardly observed. However, the surface of the activated carbon shows the characteristics of loose and porous, and the pore structure extending to the surface of the activated carbon mainly presents a circle or ellipse shape. Compared with the irregular pore structure such as mesh and crack, the circular and elliptical pore structure has better ion transport efficiency, which can reduce the resistance in the process of electrolyte ion transport.

#### XRD analysis of SACs

**Figure 7:**
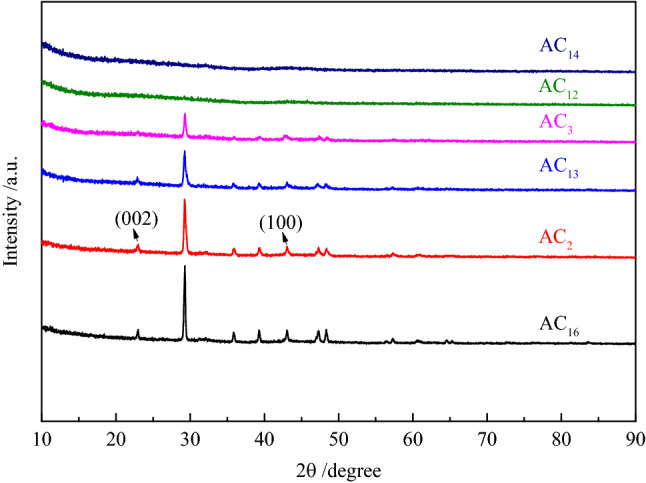
XRD patterns of SACs.

The XRD patterns of SACs are shown in Fig. 7. The XRD patterns of AC_16_, AC_2_, AC_13_ and AC_3_ demonstrate two sharp diffraction peaks at 2$\uptheta \hspace{0.17em}$= 23° and 44°, which corresponding to (002) and (100) diffractions for carbon. This indicates that the four groups of SACs have a certain graphite microcrystalline structure^46^. Activated carbons with high specific surface area usually have a relatively poor electric conductivity due to their abundant pore structures, the existence of graphite microcrystalline can greatly improve their electric conductivity, which resulted for the improvement of their electrochemical performance. The two sharpened diffraction peaks of AC_12_ and AC_14_ disappeared, indicating that the graphite microcrystalline structure in carbon materials is destroyed during chemical activation. In addition, it can be found from the XRD patterns that in addition to the diffraction peaks of graphite, there is also the presence of silica crystal peaks (2θ = 29° and 47°). The presence of silicon dioxide will not only reduce the gravimetric capacitance of activated carbon, but also increase the resistance of the supercapacitors, deteriorating the electrochemical stability of the supercapacitor.

#### The FT-IR analysis of SACs

**Figure 8:**
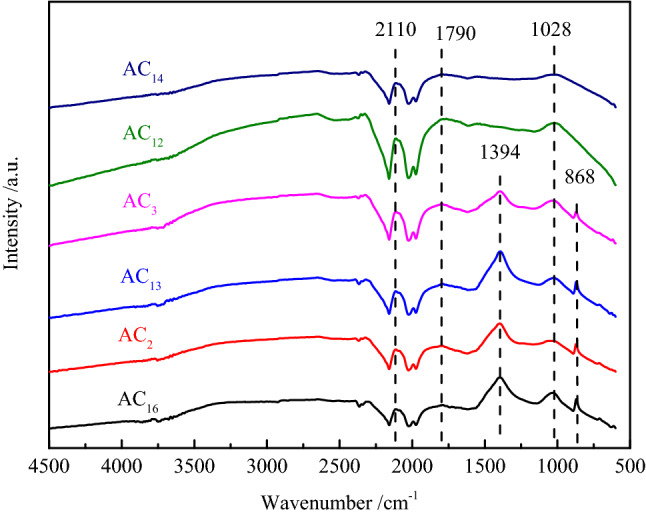
FT-IR spectrum of SACs.

The surface functional groups are associated with the electrochemical performance of the carbon materials by influencing their wettability, polarity and stability^47–51^. In addition, the existence of some surface functional groups can improve the capacitance by generating pseudocapacitance^52^. The FT-IR spectrum of SACs are shown in Fig. 8, the band is observed around the region of 1028 cm^−1^ which could be attributed to the stretching vibrations of the C–O bonds of esters, alcohols, phenols or ethers^53^. The band at 1790 cm^−1^, observed in the spectrum is attributed to the C=O stretching vibration of nonaromatic carboxyl groups with higher intensity in the spectrum resulting from the partial dehydrogenation^54^. The presence of the above two surface functional groups contributes to the improvement of the electrochemical performance of activated carbon in alkaline electrolyte. In addition, the bands at 2110 cm^−1^ in SACs are ascribed to the stretching vibration of C≡C, the existence of C≡C helps to improve the conjugation of π-bond on the surface of activated carbon, thus enhancing the electric conductivity of activated carbon^55^. The vibration peaks observed at 868 cm^−1^ and 1394 cm^−1^ are attributed to the stretching vibration of aromatic ring C–H and phenolic hydroxyl O–H, respectively.

### Electrochemical properties of SACs

#### Gravimetric capacitance of SACs

**Figure 9:**
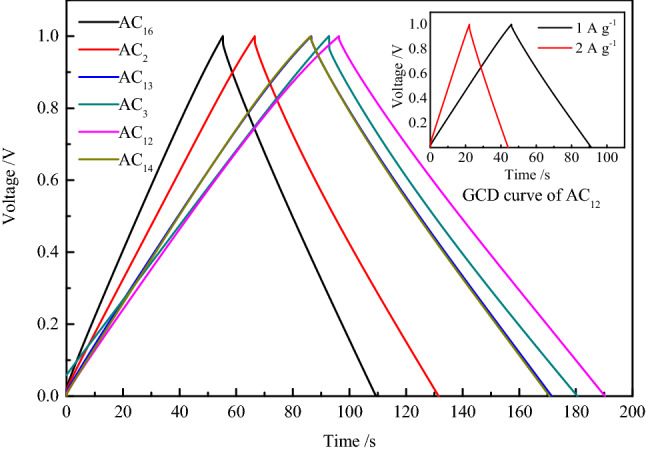
GCD curves of SACs at the current density of 0.5 A g^−1^.

The GCD curves of SACs at the current density of 0.5 A g^−1^ are shown in Fig. 9. It can be seen from the figure that all curves show good isosceles triangle shape, indicating that the supercapacitors have good double layer capacitance properties^56^. In addition, there is almost no voltage drop at the moment when the supercapacitors change from charging to discharging, which indicates that SACs have low equivalent series resistance and high charge–discharge efficiency. Even at high current density, the GCD curves of SACs still show the characteristic of standard isosceles triangle, indicating that activated carbon electrodes can provide efficient transport channel for a large number of electrolyte ions, which is beneficial to the application of activated carbon in high power conditions.

Electrode materials with larger capacitance and smaller mass have greater potential in some applications, hence, gravimetric capacitance is a very important property for carbon electrode materials^57^. To investigate the detailed capacitance performance, the gravimetric capacitance of SACs is calculated from the GCD discharge data at the current density of 0.1, 0.2, 0.5, 1, 2 and 5 A g^−1^, and the capacitance values are shown in Table 10. As can be seen from the table that the gravimetric capacitance values of SACs decay at a slow rate with the increase of current density, which indicates that SACs not only possess good gravimetric capacitance characteristics but also show excellent capacitance retention characteristics, among which the capacitance performance of the AC_12_ is the most outstanding. The capacitance value is almost as much as those of some terrestrial biomass–based activated carbons, such as corn^58^ and wheat straw^59^. The capacitance values at 0.1 A g^−1^ of biomass-based activated carbon are shown in Table 11.With the current density increases from 0.1 to 5 A g^−1^, the gravimetric capacitance retention of AC_12_ reaches up to 83.9%, and the capacitance attenuation trend decreases with the increase of current density, which indicates that the activated carbon possesses excellent capacitance performance even at high current density. The excellent capacitance retention shows that SACs have excellent rate performance, which makes the supercapacitor maintain a large gravimetric capacitance even at a large charge–discharge current density. This can not only greatly shorten the charging time of supercapacitors, but also exhibit excellent electrochemical performance in high-power applications, making supercapacitors have a wider range of applications.

**Table 10:** Gravimetric capacitance of SACs at different current densities.

Sample	Gravimetric capacitance at different current densities (F g^−1^)					
	0.1 A g^−1^	0.2 A g^−1^	0.5 A g^−1^	1 A g^−1^	2 A g^−1^	5 A g^−1^
AC_16_	157.1	147.5	142.5	132.7	131.6	123.5
AC_2_	205.2	193.0	181.4	172.3	169.6	159.3
AC_13_	239.8	226.1	216.1	210.2	208.1	205.3
AC_3_	260.6	244.9	233.6	229.9	218.7	210.3
AC_12_	264.8	249.1	237.3	233.5	227.2	222.1
AC_14_	235.5	223.0	213.9	208.9	203.9	195.2

**Table 11:** Capacitance values at 0.1 A g^−^1of biomass-based activated carbons.

Number	Materials	Activation agent	Electrolyte	SBET (m^2^ g^−1^)	Capacitance (F g^−1^)	References
1	Corn	KOH	KOH	3199	257	^58^
2	Wheat straw	KOH	MeEt_3_NBF_4_	2316	251	^59^
3	Coconut shell	KOH	KOH	3436	368	^60^
4	Potato starch	KOH	KOH	2342	335	^61^
5	Rice husk	NaOH	KOH	3969	368	^62^
6	Sunflower seed shell	KOH	KOH	2509	311	^63^
7	Sargassum	KOH	KOH	3155	265	This work

#### Cyclic voltammetry characteristics of SACs

**Figure 10:**
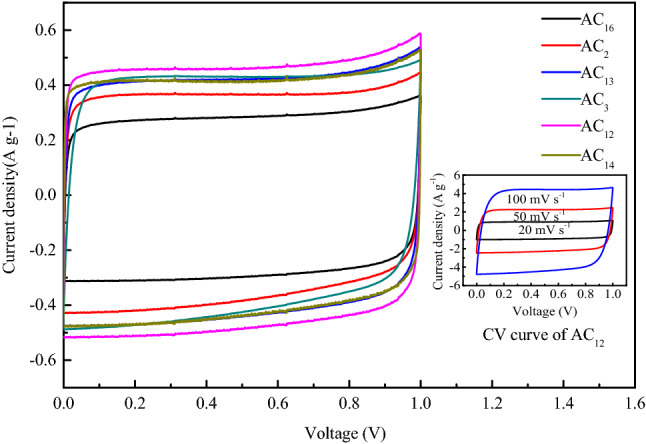
CV curves of SACs at the scan rate of 10 mV s^−1^.

**Figure 11:**
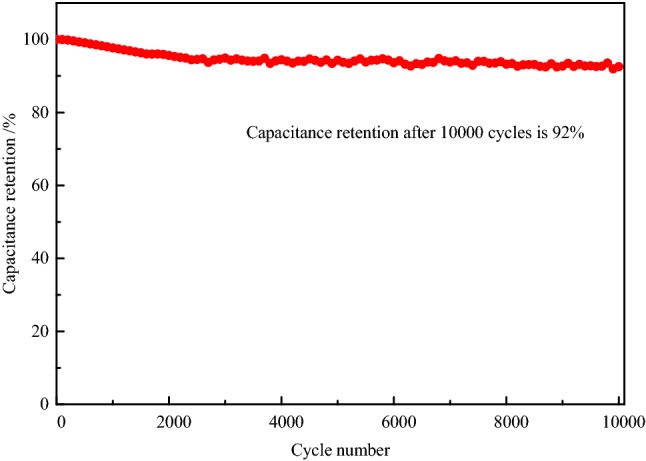
Cyclic performance of AC_12_ at current density 5 A g^−1^.

The CV curves of SACs at the scan rate of 10 mV s^−1^ are shown in Fig. 10. The CV curves of the SACs show excellent symmetry and approximate rectangular shape, which indicates that the energy storage mode of the activated carbon electrode is basically electric double layer capacitance, without the existence of pseudo capacitance. In Fig. 11, the inherent stability of activated carbon and pure electric double layer energy storage contribute to the excellent cycle stability of activated carbon. With the increase of voltage scanning rate, the gradual weakening of the rectangular characteristics of CV curve is observed. Due to the existence of the dispersed capacitance effect, the speed of current reaching the platform at the time of changing the voltage scanning direction is delayed, resulting in the disappearance of the standard rectangular characteristic of the CV curve. However, even at the voltage scanning rate of 100 mV s^−1^, the CV curve of activated carbon still shows a good rectangular characteristic, which indicates that the electrode material has a rapid ion response ability, and the pore structure of the activated carbon can meet the rapid diffusion and transmission of electrolyte ions in the carbon electrode.

The cyclic stability of activated carbon is closely related to the service life of supercapacitors, and even determines whether supercapacitors can be used in practice. Figure 11 shows the cyclic characteristics of AC_12_ at a current density of 5 A g^−1^ for 10,000 cycles. Since the energy storage in supercapacitors are highly dominated by the double layer capacitance, the charge and discharge processes are almost completely reversible. The capacitance retention rate of AC_12_ is as high as 92% after 10,000 times of constant current charge and discharge, indicating that activated carbon has excellent cycling stability. Moreover, the decay of the carbon electrode capacitance mainly occurs in the first 3000 cycles, after which the capacitance of the activated carbon remains at a relatively stable level. After 3000 cycles of charge and discharge, the irreversible reaction on the carbon electrode basically disappears, and the energy storage mode of the supercapacitor is transformed into complete electric double-layer energy storage. The absence of the conversion between electric energy and chemical energy makes the capacitance decay in the cycle process almost negligible. The capacitance decay in the second stage is mainly caused by the collapse and damage of carbon electrode pore structure during charge and discharge. The insignificant capacitance decay shows that activated carbon has good chemical stability in KOH electrolyte.

**Figure 12:**
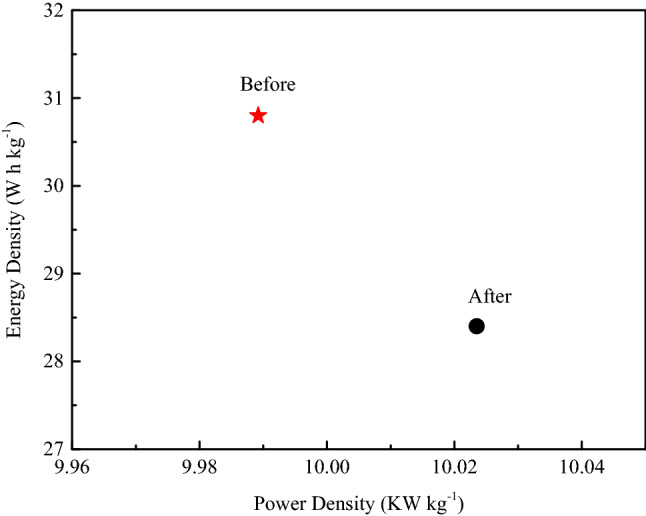
Ragone plots of the supercapacitor before and after stability test.

The energy density and power density of the supercapacitor based on AC_12_ are shown in Fig. 12. The energy density reaches 30.8 W h kg^−1^ at the power density of 9989.2 W kg^−1^, and can retain 28.4 W h kg^−1^ at the power density 10,023.5 W kg^−1^. There is only a small attenuation of the energy density after 10,000cycles.

#### Relationship between gravimetric capacitance and specific surface area of SACs

**Figure 13:**
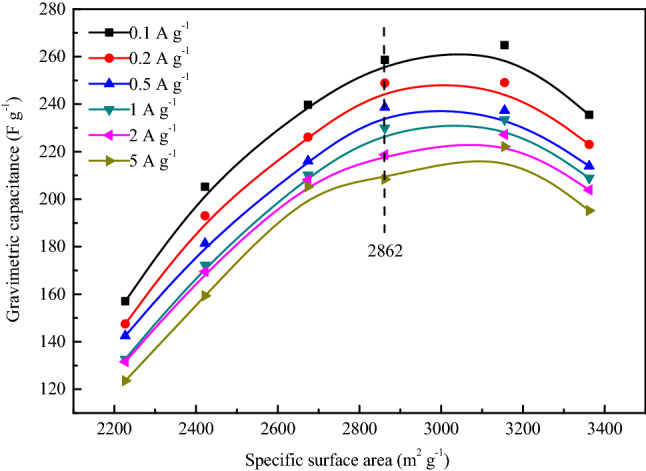
Relationship between gravimetric capacitance and *S*_*BET*_ of SACs.

The diameter of nitrogen molecule is very close to the diameter of K^+^ and OH^−^ ion in electrolyte, so the pore structure that nitrogen can reach, K^+^ and OH^−^ ion can also basically reach and form electric double layer. Therefore, the gravimetric capacitance of activated carbon is directly proportional to the specific surface area according to the electric double layer theory. However, the gravimetric capacitance of activated carbon is affected by many factors, such as pore diameter distribution, surface functional groups, graphitization degree, etc. In order to improve the gravimetric capacitance of SAC by adjusting its pore structure, the relationship between gravimetric capacitance and specific surface area was studied, the results are shown in Fig. 13.

It can be seen from the Fig. 13 that the gravimetric capacitance of AC_16_, AC_2_, AC_13_ and AC_3_ shows a good linear relationship with the specific surface area at all test current densities. This is attributed to the fact that these activated carbons have similar crystal structure, surface functional groups and pore diameter distribution. Based on this fact, with the increase of specific surface area, the effective adsorption surface area and ion transport channel of activated carbon increase linearly, resulting in a linear relationship between gravimetric capacitance and specific surface area. The linear relationship disappears with the continuous increase of specific surface area, the increase extent of gravimetric capacitance of AC_13_ decreases obviously, and the gravimetric capacitance of AC_14_ is even smaller than that of AC_13_. The absence of graphite microcrystalline structure in AC_12_ and AC_14_ is not conducive to their electrochemical performance, making the electric conductivity of AC_12_ and AC_14_ worse than that of AC_16_, AC_2_, AC_13_ and AC_3_. In addition, the micropore diameter distribution of AC_14_ is obviously different from other activated carbons. Although the pore structure of all activated carbons is dominated by micropores, the number of micropores with a diameter of 0.4 ~ 0.5 nm in AC_14_ is significantly more than that of other activated carbons. It is very difficult for the electrolyte ions to enter into this part of ultrafine micropores, which results in that this pore structure cannot provide effective adsorption surface area for electrolyte ions and further generate electric double layer capacitance.

## Conclusion

Among the measures to regulate the specific surface area of SAC, the most effective measure is to adjust the impregnation ratio, effects of activation temperature and activation time is negligible. With the increase of impregnation ratio, the specific surface area of SAC first increased and then decreased. The effect of impregnation ratio and activation temperature on the total pore volume of SAC is significant, the impregnation ratio has larger influence than activation temperature. With the increase of impregnation ratio, the total pore volume of SAC first increased and then decreased. With the increase of carbonization temperature, the total pore volume of SAC continued to increase to a large extent. The adjustment of activation temperature is mainly selected to realize the regulation of average pore diameter of SAC, with the increase of activation temperature, the average pore diameter of activated carbon always presents an increasing trend. All SACs have high specific surface area and developed pore structure dominated by micropores, and exhibit excellent electric double layer capacitance performance. AC_16_, AC_2_, AC_13_ and AC_3_ prepared in this study have similar material properties and pore diameter distribution. On this basis, the gravimetric capacitance and specific surface area show a good linear relationship.

## Data Availability

The datasets used and analyzed during the current study available from the corresponding author on reasonable request.
